# Multiple Roles of Flagellar Export Chaperones for Efficient and Robust Flagellar Filament Formation in *Salmonella*

**DOI:** 10.3389/fmicb.2021.756044

**Published:** 2021-10-06

**Authors:** Tohru Minamino, Yusuke V. Morimoto, Miki Kinoshita, Keiichi Namba

**Affiliations:** ^1^Graduate School of Frontier Biosciences, Osaka University, Suita, Japan; ^2^Department of Physics and Information Technology, Faculty of Computer Science and Systems Engineering, Kyushu Institute of Technology, Iizuka, Japan; ^3^Japan Science and Technology Agency, PRESTO, Kawaguchi, Japan; ^4^RIKEN SPring-8 Center and Center for Biosystems Dynamics Research, Suita, Japan; ^5^JEOL YOKOGUSHI Research Alliance Laboratories, Osaka University, Suita, Japan

**Keywords:** bacterial flagella, chaperone, flagellar assembly, flagellar filament, protein secretion

## Abstract

FlgN, FliS, and FliT are flagellar export chaperones specific for FlgK/FlgL, FliC, and FliD, respectively, which are essential component proteins for filament formation. These chaperones facilitate the docking of their cognate substrates to a transmembrane export gate protein, FlhA, to facilitate their subsequent unfolding and export by the flagellar type III secretion system (fT3SS). Dynamic interactions of the chaperones with FlhA are thought to determine the substrate export order. To clarify the role of flagellar chaperones in filament assembly, we constructed cells lacking FlgN, FliS, and/or FliT. Removal of either FlgN, FliS, or FliT resulted in leakage of a large amount of unassembled FliC monomers into the culture media, indicating that these chaperones contribute to robust and efficient filament formation. The ∆*flgN* ∆*fliS* ∆*fliT* (∆NST) cells produced short filaments similarly to the ∆*fliS* mutant. Suppressor mutations of the ∆NST cells, which lengthened the filament, were all found in FliC and destabilized the folded structure of FliC monomer. Deletion of FliS inhibited FliC export and filament elongation only after FliC synthesis was complete. We propose that FliS is not involved in the transport of FliC upon onset of filament formation, but FliS-assisted unfolding of FliC by the fT3SS becomes essential for its rapid and efficient export to form a long filament when FliC becomes fully expressed in the cytoplasm.

## Introduction

**Figure 1:**
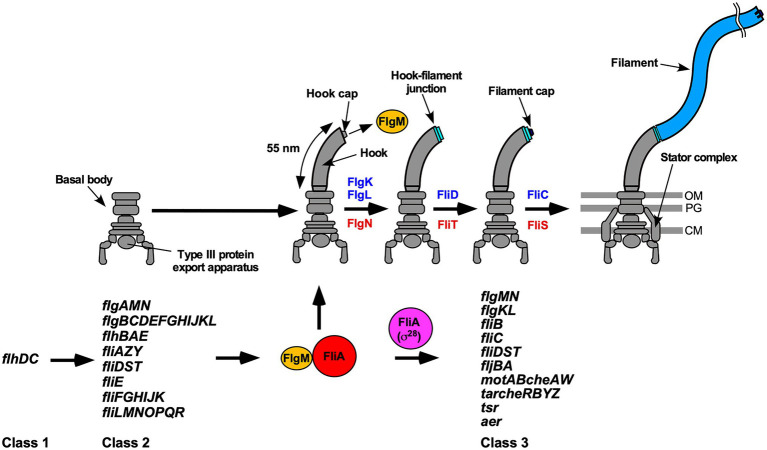
Flagellar assembly and hierarchical flagellar gene expression. Flagellar assembly begins with the basal body, followed by the hook (FlgE) with the help of the hook cap (FlgD). Upon completion of hook assembly, the hook cap is replaced by FlgK. FlgK and FlgL self-assemble at the hook tip in this order to form the hook-filament junction structure. Then, FliD forms the filament cap at the tip of the junction and promotes the assembly of FliC into the long helical filament. During hook-basal body (HBB) assembly, FlhD and FlhC, which are class 1 gene products, act as a transcriptional activator to induce the transcription of class 2 genes required for HBB formation. When the hook length reaches its mature length of 55nm in *Salmonella*, the flagellar type III secretion system (fT3SS) switches its substrate specificity from rod- and hook-type proteins to filament-type ones, thereby terminating hook assembly and filament assembly at the hook tip. As a result, FlgM is secreted *via* the fT3SS into the culture media, and so FliA acts as a flagellum-specific sigma factor (σ^28^) to transcribe class 3 genes encoding proteins required for filament formation, motility, and chemotaxis. FlgN, FliT, and FliS act as flagellar export chaperones specific for FlgK/FlgL, FliD, and FliC, respectively. These chaperones facilitate the docking of their substrates to the fT3SS for efficient and rapid protein unfolding and export. Except for the *fliC* gene, the *flgKL*, *flgMN*, and *fliDST* genes are transcribed from both class 2 and class 3 promoters. OM, outer membrane; PG, peptidoglycan layer; CM, cytoplasmic membrane.

Many pathogenic bacteria utilize flagella to swim in viscous fluids to reach and attach to host cells for effective infection and colonization. The flagellum of *Salmonella enterica* serovar Typhimurium (thereafter referred to as *Salmonella*) is composed of the basal body, which acts as a rotary motor fueled by proton motive force (PMF) across the cell membrane, the filament, which functions as a helical propeller to produce the thrust that drives swimming motility and the hook connecting the basal body and filament to act as a universal joint. Flagellar assembly begins with the basal body, followed by the hook (FlgE) with the help of the hook cap (FlgD). After completion of hook assembly, the hook cap is replaced by FlgK, and 11 FlgK and FlgL subunits are assembled to form the hook-filament junction structure at the hook tip to start filament assembly. Five FliD molecules self-assemble into the filament cap at the tip of the junction structure, and finally, tens of thousands of flagellin molecules (FliC or FljB) polymerize into the long helical filament with the help of the filament cap (Figure 1; Minamino and Namba, 2004; Nakamura and Minamino, 2019).

To build the flagella beyond the inner and outer membranes, flagellar building blocks are translocated across the cell membrane *via* the flagellar type III secretion system (fT3SS), diffuse down the central channel of the growing flagellar structure, and assemble at the distal end. The fT3SS consists of a PMF-driven transmembrane export gate complex made of FlhA, FlhB, FliP, FliQ, and FliR and a cytoplasmic ATPase complex composed of FliH, FliI, and FliJ (Minamino, 2014; Minamino et al., 2020b). Because the cytoplasmic ATPase ring complex is dispensable for flagellar protein export, the export gate complex works not only as a PMF-driven protein transporter but also as a PMF-driven unfoldase (Minamino and Namba, 2008; Paul et al., 2008; Terashima et al., 2018, 2020). In addition, FlgN, FliA, FliS, and FliT act as cytoplasmic export chaperones specific for FlgK/FlgL, FlgM, FliC, and FliD, respectively (Fraser et al., 1999; Auvray et al., 2001; Aldridge et al., 2006).

In *Salmonella*, flagellar genes are divided into three classes according to their transcriptional hierarchy (Figure 1; Kutsukake et al., 1990). Class 1 genes, *flhD* and *flhC*, encode a positive regulator required for the transcription of class 2 genes. The class 2 genes encode proteins required for the structure and assembly of hook-basal body (HBB). FliA is a class 2 gene product and acts as the flagellum-specific sigma factor (σ^28^) that transcribes class 3 genes encoding proteins required for filament formation, motility, and chemotaxis (Ohnishi et al., 1990). Such transcriptional coordination ensures that flagellar building blocks, motor proteins, and proteins involved in chemotactic signaling are expressed in different stages during flagellar assembly (Chevance and Hughes, 2008). First, the fT3SS transports six rod-type (FliE, FlgB, FlgC, FlgF, FlgG, and FlgJ) and three hook-type (FlgD, FlgE, and FliK) proteins to produce the HBB (Minamino, 2018). At this stage, four filament-type proteins (FlgK, FlgL, FlgM, FliD) and four export chaperones (FlgN, FliA, FliS, FliT) are also expressed along with other class 2 gene products (Figure 1). However, the fT3SS does not transport these filament-type proteins until HBB assembly is complete (Minamino et al., 1999). FlgM functions as an anti-sigma factor to suppress the σ^28^ activity of FliA during HBB assembly (Ohnishi et al., 1992). When the hook reaches its mature length of about 55nm, the fT3SS switches its substrate specificity from rod- and hook-type proteins to filament-type ones, thereby terminating HBB assembly and initiating filament formation (Kutsukake et al., 1994a). As a result, FlgM is secreted *via* the fT3SS into the culture media, allowing FliA to act as σ^28^ to transcribe the *fliC* gene to start forming the filament at the hook tip (Figure 1; Hughes et al., 1993; Kutsukake, 1994). Thus, the fT3SS appears to coordinate gene expression with assembly. In addition, the FlgN, FliS, and FliT chaperones also regulate the expression levels of class 2 and 3 genes to control the number of flagella per cell (Yokoseki et al., 1996; Karlinsey et al., 2000a; Aldridge et al., 2010; Xu et al., 2014).

FlgN, FliS, and FliT adopt highly α-helical structures (Evdokimov et al., 2003; Imada et al., 2010; Kinoshita et al., 2016). They bind to the intrinsically disordered C-terminal region of their cognate substrates not only to suppress their premature aggregation and proteolysis in the cytoplasm (Fraser et al., 1999; Bennett et al., 2001; Aldridge et al., 2003; Ozin et al., 2003) but also to efficiently transfer their substrates to the fT3SS export gate (Thomas et al., 2004; Bange et al., 2010; Imada et al., 2010; Minamino et al., 2012a,b; Kinoshita et al., 2013). Interactions between the flagellar chaperones and their cognate substrates induce rearrangements of helices in the chaperone structures to regulate the affinities for their binding partners during the protein export process (Thomas et al., 2004; Evans et al., 2006; Bange et al., 2010; Imada et al., 2010; Kinoshita et al., 2013, 2016; Khanra et al., 2016).

The C-terminal cytoplasmic domain of FlhA (FlhA_C_) forms a ring-like structure in the fT3SS as the docking platform for the export substrates and ensures the strict order of flagellar protein export (Abrusci et al., 2013; Terahara et al., 2018; Inoue et al., 2019, 2021; Minamino et al., 2012a). The FlgN, FliS, and FliT chaperones in complex with their cognate substrates bind to FlhA_C_ with nanomolar affinity (Bange et al., 2010; Minamino et al., 2012a; Kinoshita et al., 2013; Xing et al., 2018). Highly conserved tyrosine residues of chaperones, Tyr-122 of FlgN, Tyr-10 of FliS, and Tyr-106 of FliT, are critical for the interaction with FlhA_C_ at its specific binding pocket (Minamino et al., 2012a; Kinoshita et al., 2013; Altegoer et al., 2018; Xing et al., 2018). The FlgN(Y122A) and FliS(Y10S) substitutions reduce the secretion levels of FlgK/FlgL and FliC, respectively, suggesting that the flagellar chaperones promote the docking of their cognate substrates to the FlhA_C_ ring and that the interaction of these chaperones with FlhA_C_ assists efficient protein unfolding and transport by the transmembrane export gate complex (Minamino et al., 2012a; Kinoshita et al., 2013; Furukawa et al., 2016).

FlgN and FliT bind to FliI and FliJ, whereas FliS does not (Thomas et al., 2004; Evans et al., 2006; Bange et al., 2010; Imada et al., 2010; Minamino et al., 2012b; Sajó et al., 2014). This distinct characteristic of FliS would be related to the fact that the number of FliC subunits per filament is three orders of magnitude higher than that of FlgK, FlgL, and FliD subunits (Minamino and Namba, 2004). Because FliI and FliJ bind to FlhA_C_, it has been proposed that interactions of FlgN and FliT with FliI and FliJ provide an advantage for efficient docking of FlgK, FlgL, and FliD to the FlhA_C_ ring prior to the export of a largely excessive amount of FliC molecules (Evans et al., 2006; Bange et al., 2010; Minamino et al., 2016; Inoue et al., 2018). However, it remains unclear how these chaperones determine the substrate export order for efficient flagellar filament assembly.

*Salmonella* cells lacking FliS (thereafter referred to as ∆S, Table 1) produce very short filaments with their lengths shorter than 3μm (Yokoseki et al., 1995). Extragenic suppressor mutations in FliC allow the ∆S cells to produce longer flagellar filaments (Furukawa et al., 2016). These suppressor mutations significantly destabilize the folded structure of FliC monomer. Furthermore, they do not affect the binding affinity of FliC for FlhA_C_ at all (Furukawa et al., 2016). These observations suggest that the PMF-driven unfolding step of FliC monomer by the transmembrane export gate complex limits the export rate of FliC in the ∆S mutant (Furukawa et al., 2016). However, it remains unknown why the filament growth of the ∆S mutant stops at short lengths and how FliS supports the export gate complex to facilitate FliC unfolding for its efficient transport to form a long filament.

**Table 1:** Strains and plasmids used in this study.

*Salmonella* strains	Abbreviated name	Relevant characteristics	Source or reference
SJW1103	WT	Wild type for motility and chemotaxis	Yamaguchi et al., 1984
SJW2177	∆K	*flgK*	Homma et al., 1984
MM9001	∆N	∆*flgN*::*tetRA*	Minamino et al., 2012a
MM9101	∆S	∆*fliS*::*km*	This study
MM9102	∆S/P*_araBAD_*-*fliS*	∆*fliS*::*km* ∆*araBAD*::*fliS*	This study
MM9103	∆S/P*_araBAD_*-*fliS* T-POP	∆*fliS*::*km* ∆*araBAD*::*fliS* P*_flhDC_*::T-POP (DEL-25)	This study
MM9104	∆T	∆*fliT*::*km*	This study
MM9105	∆NS	∆*flgN*::*tetRA* ∆*fliS*::*km*	This study
MM9106	∆NT	∆*flgN*::*tetRA* ∆*fliT*::*km*	This study
MM9107	∆ST	∆*fliS-fliT*::*km*	This study
MM9108	∆NST	∆*flgN*::*tetRA* ∆*fliS-fliT*::*Km*	This study
MMC9108-1	∆NST *fliC*(∆195–274)	∆*flgN*::*tetRA* ∆*fliS-fliT*::*km fliC*(∆195–274)	This study
MMC9108-2	∆NST *fliC*(R92S)	∆*flgN*::*tetRA* ∆*fliS-fliT*::*km fliC*(R92S)	This study
MMC9108-3	∆NST *fliC*(∆245–289)	∆*flgN*::*tetRA* ∆*fliS-fliT*::*km fliC*(∆245–289)	This study
MMC9108-4	∆NST *fliC*(E153A)	∆*flgN*::*tetRA* ∆*fliS-fliT*::*km fliC*(E153A)	This study
MMC9108-5	∆NST *fliC*(Q128R)	∆*flgN*::*tetRA* ∆*fliS-fliT*::*km fliC*(Q128R)	This study
MM9109	∆M	∆*flgM*::*km*	This study

To clarify the role of the flagellar export chaperones in filament assembly, we constructed flagellar chaperone-deficient mutants and analyzed their efficiency of filament formation. We show that removal of either FlgN, FliS, or FliT causes leakage of a significantly larger amount of unassembled FliC monomers into the culture media compared to the *Salmonella* wild-type strain (hereafter referred to as WT), suggesting that these chaperones not only assist the export of their cognate substrates but also contribute to efficient and robust filament formation. We also show that FliS-assisted unfolding of FliC by the export gate complex becomes essential for rapid and efficient export of FliC to form a long filament after FliC synthesis is complete.

## Materials and Methods

### *Salmonella* Strains, Transductional Crosses, and DNA Sequencing

*Salmonella* strains used in this study are listed in Table 1. To isolate spontaneous pseudorevertants from the *Salmonella* ∆*flgN* ∆*fliS* ∆*fliT* cells, 50-μl overnight cultures were streaked out on soft agar plates [1% (w/v) tryptone, 0.5% (w/v) NaCl, 0.35% (w/v) Bacto agar], and the plates were incubated at 30°C for 2days to look for swarms emerging from each streak. Six motile colonies were isolated from such swarms. P22-mediated transductional crosses were carried out with p22HT*int* (Schmiger, 1972). DNA sequencing reactions were carried out using BigDye v3.1 (Applied Biosystems), and the reaction mixtures were analyzed by a 3,130 Genetic Analyzer (Applied Biosystems).

#### Motility Assay

Fresh colonies were inoculated onto soft agar plates and incubated at 30°C. At least seven independent colonies of each mutant strain were analyzed.

#### Secretion Assay

*Salmonella* cells were grown in L-broth [1% (w/v) tryptone, 0.5% (w/v) yeast extract, 0.5% (w/v) NaCl] at 30°C with shaking until the cell density had reached an OD_600_ of ca. 1.2–1.4, and then, each culture was heated at 65°C for 5min to depolymerize the filaments into flagellin monomers, followed by centrifugation (8,000×*g*, 5min, 4°C) to obtain the cell pellet and supernatant separately. Proteins in whole cellular and culture supernatant fractions were normalized to the OD_600_ unit of each culture to give a constant number of *Salmonella* cells. Each cell pellet was resuspended in SDS-loading buffer [62.5mM Tris-HCl, pH 6.8, 2% sodium dodecyl sulfate (SDS), 10% glycerol, 0.001% bromophenol blue] containing 1μl of 2-mercaptoethanol. Proteins in the culture supernatants were precipitated by 10% trichloroacetic acid (TCA), suspended in a Tris-SDS loading buffer (one volume of 1M Tris, nine volumes of SDS-loading buffer) containing 1μl of 2-mercaptoethanol and heated at 95°C for 3min. After SDS-polyacrylamide gel electrophoresis (SDS-PAGE), immunoblotting with polyclonal anti-FlgK, anti-FlgL or anti-FliD antibody was carried out as described previously (Minamino and Macnab, 1999). Detection was performed with Amersham ECL Prime western blotting detection reagent (Cytiva). Chemiluminescence signals were captured by a Luminoimage analyzer LAS-3000 (GE Healthcare). At least three independent experiments were performed.

#### Observation of Negatively Stained *Salmonella* Cells by Electron Microscopy

*Salmonella* cells were exponentially grown in 5ml of L-broth at 30°C. A 5μl of each cell culture was applied to a carbon-coated copper grid and negatively stained with 0.5% (w/v) phosphotungstic acid, pH 6.5. Micrographs were recorded at a magnification of ×1,200 with a JEM-1011 transmission electron microscope (JEOL) operating at 100kV.

#### FliC Leakage Measurements During Flagellar Filament Assembly

*Salmonella* cells were grown in 5ml of L-broth at 30°C with very gentle shaking to avoid mechanical shearing of flagellar filaments until the cell density had reached an OD_600_ of ca. 1.2–1.4.

To prepare total extracellular FliC subunits (filaments attached to *Salmonella* cell bodies and unassembled FliC monomers secreted into the culture media), a 1.5ml of culture was heated at 65°C for 5min to depolymerize the filaments into FliC monomers, followed by centrifugation (8,000×*g*, 5min, 4°C) to obtain the cell pellet and culture supernatant separately. Proteins in the culture supernatant were precipitated by 10% TCA, suspended in a Tris-SDS loading buffer, and heated at 95°C for 3min.

To prepare polymerized FliC subunits (filaments attached to the cell bodies), a 1.5ml of culture was centrifuged (8,000×*g*, 5min, 4°C), and the cell pellet and culture supernatant were collected separately. The cell pellet was suspended in a 1.5ml of PBS (8g of NaCl, 0.2g of KCl, 3.63g of Na_2_HPO_4_ 12H_2_O, 0.24g of KH_2_PO_4_, pH 7.4 per liter) and heated at 65°C for 5min, followed by centrifugation (8,000×*g*, 5min, 4°C) to obtain the cell pellet and supernatant, which contained cytoplasmic FliC molecules and depolymerized FliC monomers, respectively. Depolymerized FliC monomers in the supernatant were precipitated by 10% TCA, suspended in a Tris-SDS loading buffer, and heated at 95°C for 3min.

To prepare FliC monomers leaked out into the culture media during filament assembly, the culture supernatant was ultracentrifuged at 85,000×*g* for 1h at 4°C, and the pellet and supernatant, which contained flagellar filaments detached from the cell bodies during shaking culture and FliC monomers leaked out into the culture media, respectively, were collected separately. FliC monomers in the supernatant were precipitated by 10% TCA, suspended in the Tris/SDS loading buffer and heated at 95°C for 3min.

Samples were analyzed by SDS-PAGE with Coomassie Brilliant Blue (CBB) staining. Gel images were captured by a Luminoimage analyzer LAS-3000 (GE Healthcare). The band intensity was analyzed using an image analysis software, CS Analyzer 4 (ATTO, Tokyo, Japan). Three independent measurements were performed.

#### Tetracycline-Induced Secretion Assay

*Salmonella* ∆*fliS* ∆*araBAD*::*fliS* cells with a T-POP insertion, which is located between the *flhDC* promoter and the transcription start site (Karlinsey et al., 2000b), were grown overnight in L-broth at 30°C and diluted 100-fold into 25ml of fresh L-broth. When the cell density reached an OD_600_ of 0.5, tetracycline and arabinose was added to a final concentration of 15μg/ml and 0.2% (w/v), respectively. Samples were taken at 0, 15, 30, 45, 60, 90, 120, and 180min after tetracycline addition and heated at 65°C for 5min. After centrifugation (8,000×*g*, 5min, 4°C), proteins in the culture supernatant fraction were prepared by TCA. After SDS-PAGE, immunoblotting with polyclonal anti-FliC or anti-FlgM antibody was carried out. The band intensity of each blot was analyzed using an image analysis software, CS Analyzer 4 (ATTO, Tokyo, Japan). Four independent measurements were carried out.

### Observation of Flagellar Filaments

A 50μl of overnight culture of *Salmonella* wild-type and ∆*fliS* ∆*araBAD*::*fliS* cells were inoculated into fresh 5ml of L-broth and grown at 30°C with shaking until the cell density had reached an OD_600_ of *ca*. 0.5. After addition of arabinose at a final concentration of 0.2%, 100μl of the culture was collected at 1, 3, 6, 10, and 14h and washed with motility buffer (10mM potassium phosphate, 0.1mM EDTA, pH 7.0). In the experiment using a T-POP insertion, *Salmonella* ∆*fliS* ∆*araBAD*::*fliS* cells with the T-POP insertion were grown in L-broth at 30°C for 3h, and then, tetracycline and arabinose were added at a final concentration of 15μg/ml and 0.2% (w/v), respectively. The cells were collected at the indicated time intervals and washed with the motility buffer. The cells were attached to a cover slip (Matsunami glass, Japan), and unattached cells were washed away with motility buffer. Then, flagellar filaments were labelled with Alexa Fluor 594 (Invitrogen) as described before (Minamino et al., 2014). Epifluorescence of Alexa Fluor 594 was observed by an inverted fluorescence microscope (IX-83, Olympus) with a 100× oil immersion objective lens (UPLSAPO100XO, NA 1.4, Olympus) and a sCMOS camera (Prime 95B, Photometrics). Fluorescence images were analyzed using ImageJ software version 1.53 (National Institutes of Health). Statistical analyses were done using Prism 9 software (GraphPad). Comparisons were performed using a two-tailed Student’s *t*-test. A value of *p*<0.05 was considered to be statistically significant difference.

## Results

### Effect of FliS Deletion on Motility of the ∆*flgN* Mutant

A *Salmonella* ∆*flgN* mutant (thereafter referred to as ∆N, Table 1) cannot efficiently transport FlgK and FlgL to the hook tip to form the hook-filament junction structure (Bennett et al., 2001; Kutsukake et al., 1994b), and so only about 35% of the ∆N cells produce a single flagellar filament and generate a small motility ring on soft agar plates (Supplementary Figure 1A; Minamino et al., 2021). Loss-of-function mutations of either ClpXP, FlgM, or FliT increase the expression levels of FlgK and FlgL, thereby significantly increasing the probability of hook-filament junction formation even in the absence of FlgN (Aldridge et al., 2003). Removal of FliS increases the secretion level of FlgM, which in turn increases the expression level of class 3 genes (Yokoseki et al., 1996), raising the possibility that FliS deletion also increases the probability of filament assembly in the absence of FlgN. To investigate this possibility, we constructed the ∆N mutant containing the Δ*fliS*::*km* allele (thereafter referred to as ∆NS, Table 1) or the Δ*fliT*::*km* allele (thereafter referred to as ∆NT, Table 1). In agreement with a previous result, removal of FliT restored motility of the ∆N mutant to a considerable degree (Supplementary Figure 1A). As expected, the cytoplasmic levels of FlgK and FlgL were higher in the ∆NS and ∆NT mutants than the ∆N mutant (Supplementary Figure 1B, left panels, second and third rows). Deletion of FliS increased the secretion levels of FlgK and FlgL by the ∆N mutant in a way similar to FliT deletion (Supplementary Figure 1B, right panels, second and third rows). These observations suggest that the over-expression of FlgK and FlgL caused by removal of either FliS or FliT compensates for the lack of FlgN chaperone activity.

**Figure 2:**
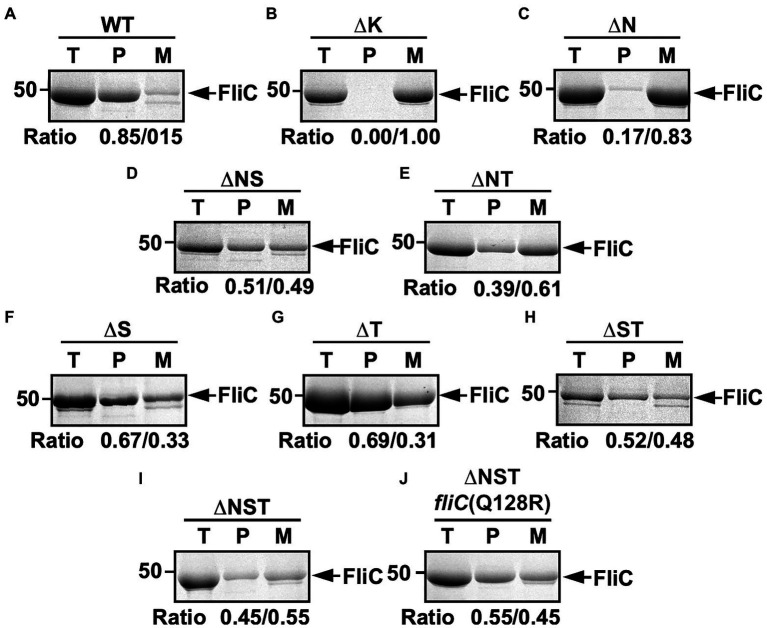
Effect of removal of flagellar export chaperones on FliC leakage during filament assembly. Measurements of FliC monomers leaked out into the culture media. CBB-staining SDS gels of total extracellular FliC proteins (indicated as T), polymerized FliC subunits in the filament (indicated as P), and FliC monomers leaked into the culture media (indicated as M) of **(A)** wild-type (WT), **(B)** *flgK* (∆K), **(C)** ∆*flgN* (∆N), **(D)** ∆*flgN* ∆*fliS* (∆NS), **(E)** ∆*flgN* ∆*fliT* (∆NT), **(F)** ∆*fliS* (∆S), **(G)** ∆*fliT* (∆T), **(H)** ∆*fliS* ∆*fliT* (∆ST), **(I)** ∆*flgN* ∆*fliS* ∆*fliT* (∆NST), and **(J)** ∆*flgN* ∆*fliS* ∆*fliT fliC*(Q128R) [∆NST *fliC*(Q128R)]. The position of 50kDa molecular mass marker is indicated on the left. The band densities of polymerized FliC subunits and FliC monomers leaked into the culture media were normalized by the total extracellular FliC level in each strain, and then, the relative ratio of the polymerized and secreted FliC subunits was calculated. We performed three independent measurements.

To investigate how deletion of FliS or FliT affects the probability of filament formation by the ∆N mutant, we isolated polymerized FliC subunits in the filament form and unassembled FliC monomers leaked into the culture media separately and analyzed them by SDS-PAGE with CBB staining. In the wild-type cells, about 85% of FliC subunits assembled into the filaments, and the remaining 15% existed as monomer in the culture supernatant (Figure 2A). In the ∆N mutant, more than 80% of FliC subunits existed as monomer in the culture supernatant (Figure 2C), indicating that the ∆N mutant cannot efficiently form the hook-filament junction at the hook tip. Additional deletion of FliS or FliT partially suppressed FliC leakage by the ∆N mutant (Figures 2D,E). Because the lack of the hook-filament junction structure causes complete leakage of unassembled FliC monomers into the culture media without filament formation (Figure 2B; Homma et al., 1984), this suggests that the ∆NS and ∆NT cells form the junction structure even in the absence of FlgN. However, a much larger amount of FliC still existed as monomer in the culture supernatant (Figures 2D,E), indicating that the ∆NS and ∆NT mutants are not able to form the hook-filament junction so efficiently. Interestingly, deletion of either FliS or FliT alone or both also increased the amount of unassembled FliC monomers leaking out into the culture supernatant even in the presence of FlgN (Figures 2F–H). Because FliC leakage by the WT cells into the culture media was minimal during filament assembly (Figure 2A), the flagellar export chaperones are required for efficient and robust filament formation.

### Effect of Flit Deletion on FliD Export

FliT is required for efficient FliD export for its assembly as the filament cap to promote FliC assembly to form a long filament as the helical propeller. However, motility of the ∆*fliT* mutant (thereafter referred to as ∆T, Table 1) was worse than the WT only slightly and almost the same as the ∆*flgM* mutant (thereafter referred to as ∆M; Supplementary Figure 2A). To clarify why FliT deletion does not inhibit motility so significantly, we compared the cytoplasmic and secretion levels of FliD between the ∆S, ∆T, and ∆M mutants (Supplementary Figure 2B). Deletion of either FliS, FliT, or FlgM increased the cytoplasmic level of FliD considerably compared to the WT (Supplementary Figure 2B, second row, left panel). This is consistent with the fact that FliT also acts as a negative regulator in the flagellar regulon so that removal of FliT results in a considerable increase in the expression level of class 3 genes, similar to the ∆M mutant (Yokoseki et al., 1996). However, the secretion level of FliD by the ∆T mutant was lower than that by the ∆S and ∆M mutants (Supplementary Figure 2B, second row, right panel), confirming that FliT is required for efficient FliD transport. Thus, the over-expression of FliD caused by removal of FliT compensates for the lack of FliT chaperone activity to some level, thereby allowing the ∆T mutant to produce long helical filaments.

### Effect of Removal of all Three Flagellar Export Chaperones on Flagellar Filament Formation

It has been reported that the ∆S and ∆*fliS* ∆*fliT* mutants (thereafter referred to as ∆ST, Table 1) produce shorter flagellar filaments than WT (Supplementary Figure 1C; Yokoseki et al., 1995). To investigate whether the ∆NS mutant also produces short flagellar filaments, we negatively stained the ∆NS cells and observed their filaments by electron microscopy (Supplementary Figure 1C). As expected, the ∆NS cells produced short filaments. Interestingly, the ∆NT mutant, of which motility was better than the ∆NS mutant but worse than the ∆T mutant (Supplementary Figure 1A), produced long filaments in a way similar to the WT and ∆T cells (Supplementary Figure 1C). Therefore, we conclude that FliS is directly involved in efficient elongation of the flagellar filament.

**Figure 3:**
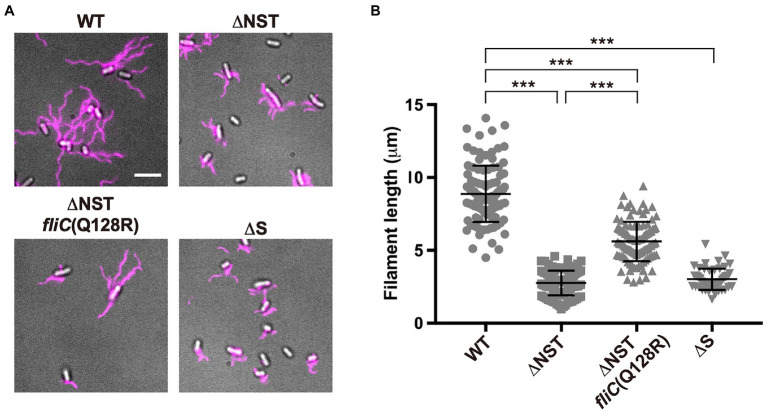
Effect of deletion of FliS on filament length. **(A)** Fluorescent images of the WT, ∆NST, ∆NST *fliC*(Q128R), and ∆S cells. The cells were grown in L-broth until the cells reached the stationary phase, and then, flagellar filaments were labelled with a fluorescent dye, Alexa Fluor 594. The fluorescence images of the filaments labelled with Alexa Fluor 594 (magenta) were merged with the DIC images of the cell bodies. Scale bar, 5.0μm. **(B)** Scatter plots of flagellar filament length. Filament length is the average of 50 filaments, and vertical lines are standard deviations. Comparisons between datasets were performed using a two-tailed Student’s *t*-test. A value of *p*<0.05 was considered to be statistically significant difference. ^***^, *p*<0.001 (also see Supplementary Table 1).

To address why the over-expression of FliC caused by removal of FliS cannot compensate for the lack of FliS chaperone activity, we constructed the ∆*flgN* ∆*fliS* ∆*fliT* mutant (Supplementary Figures 1A,B; thereafter referred to as ∆NST, Table 1) and analyzed the filament length of the ∆NST cells (Figure 3 and Supplementary Table 1). All the WT cells produced long filaments with an average length of 8.9±1.9μm (*n*=50). In contrast, 90.0 and 85.1% of the ∆S and ∆NST cells produced the filaments of shorter lengths, with an average length of 3.0±0.7μm (*n*=50) and 2.8±0.8μm (*n*=50), respectively, which are almost three times shorter than the wild-type length. This suggests that removal of FliS alone not only reduces the probability of filament formation slightly but also markedly inhibits the elongation of the filament structure. Because the ∆NST mutant displayed a short filament phenotype in a way similar to the ∆S mutant, we conclude that FliS is the most important chaperone for efficient FliC export and assembly to form a long filament.

### Isolation of Pseudorevertants From the ∆NST Mutant

**Figure 4:**
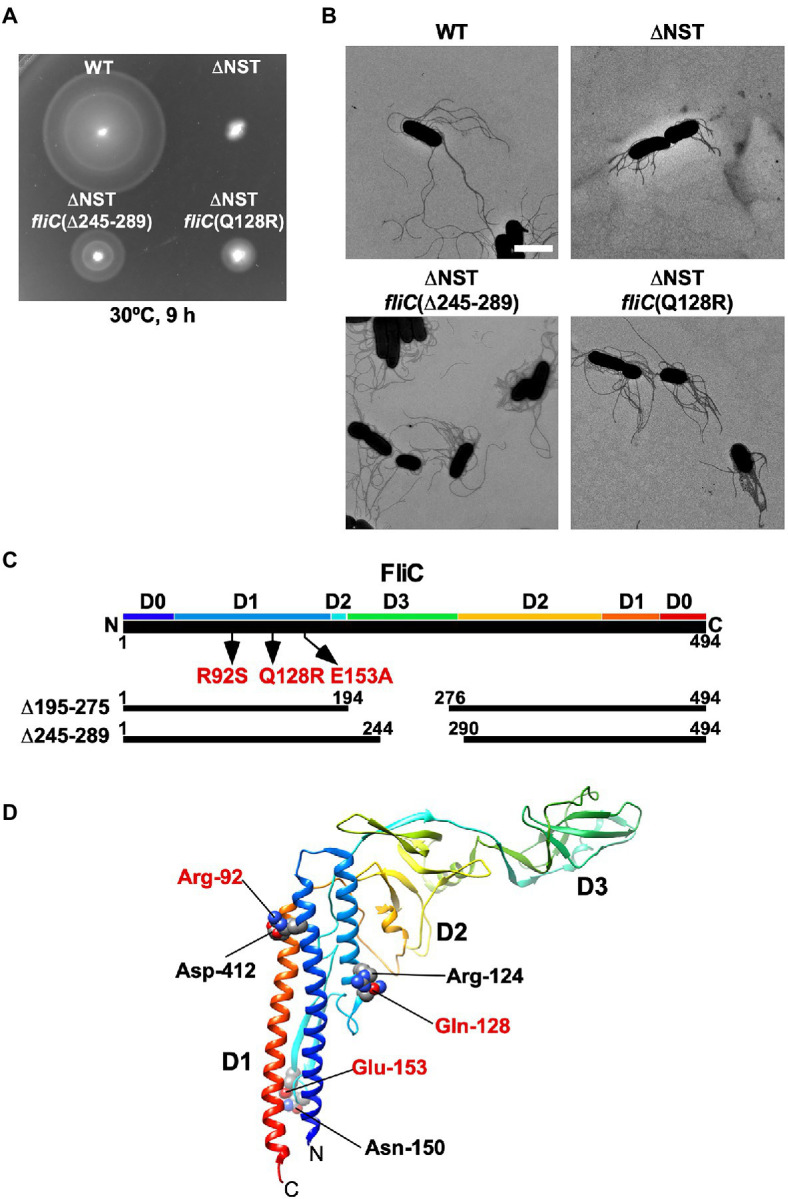
Isolation of pseudorevertants from the ∆NST mutant. **(A)** Motility of WT, ∆NST and its bypass mutants, ∆NST *fliC*(∆245–289) and ∆NST *fliC*(Q128R). Plates were incubated at 30°C for 9h. **(B)** Electron micrographs of WT, ∆NST, ∆NST *fliC*(∆245–289), and ∆NST *fliC*(Q128R) cells. Cells were negatively stained with 0.5% (w/v) phosphotungstic acid (pH 6.5). Micrographs were taken at a magnification of ×1,200. Scale bar, 2μm. **(C)** Locations of bypass mutations in FliC. FliC consists of four domains, D0, D1, D2, and D3. The point mutations in FliC are indicated by arrows. In-frame deletion variants of FliC lacking residues 195–274 and residues 245–289 are shown by solid lines. **(D)** Location of mutated residues in the atomic structure of the F41 fragment of *Salmonella* FliC (PDB ID: 1IO1). The F41 fragment of FliC, which lacks N-terminal 52 and C-terminal 44 residues, consists of domains D1, D2, and D3. The Cα backbone is color-coded from blue to red, going through the rainbow colors from the N- to the C-terminus. Arg-92, Gln-128, and Glu-153 interact with Asp-412, Arg-124, and Asn-150, respectively, to stabilize domain D1 of FliC.

We found that the ∆S, ∆ST, and ∆NST mutants leaked more unassembled FliC monomers into the culture media than the WT cells (Figure 2). Furthermore, we also found that about 10 and 15% of the ∆S and ∆NST cells produced no visible filaments (Supplementary Table 1). These observations raised the possibility that the short filament phenotype of these mutants may be a consequence of inefficient FliC polymerization rather than inefficient FliC export. To investigate this possibility, we isolated six bypass mutants from the ∆NST mutant (Figure 4). Motility of these pseudorevertants, as illustrated by MMC9108-3 [∆NST *fliC*(∆245–289)] and MMC9108-5 [∆NST *fliC*(Q128R)], was better than that of the ∆NST mutant although not at the level of the WT strain (Figure 4A). The filaments produced by these bypass mutants were longer than those of the parental ∆NST mutant (Figure 4B). Consistently, the fraction of FliC assembled into the filament was larger in the bypass mutants than the ∆NST mutant (Figures 2I,J), indicating that the filament elongation process became more efficient by these bypass mutations even in the absence of FliS.

P22-mediated transduction experiments showed that all the bypass mutations were located within the *fliC* gene. Therefore, we sequenced the *fliC* gene of these bypass mutants. They can be divided into two categories (Figure 4C). The first category consists of missense mutations: R92S, Q128R, and E153A. The other category consists of two in-frame deletions: residues 195–275 (isolated twice) and residues 245–289. We mapped the point mutations on the atomic model of FliC core fragment consisting of three domains, D1, D2, and D3 (Samatey et al., 2001). It has been reported that the FliC(R92S) and FliC(Q128R) mutations induce a conformational change of domain D1, thereby destabilizing the entire fold of FliC (Furukawa et al., 2016). Because Glu-153 interacts with Asn-150 to stabilize the β-hairpin in domain D1, we assume that the FliC(E153A) substitution may affect this β-hairpin structure, thereby destabilizing domain D1. Domain D3 of FliC largely determines the thermal stability of FliC monomer (Muskotál et al., 2010). Consistently, deletion of either residues 195–233 or residues 219–243 in domain D3, which can overcome the loss of FliS chaperone activity, destabilizes the entire structure of FliC monomer (Furukawa et al., 2016). FliS binds to the extreme C-terminal region of FliC (Ozin et al., 2003), indicating that FliC synthesis is complete prior to export. Because an interaction between FliS and FlhA_C_ is required for efficient FliC export (Bange et al., 2010; Kinoshita et al., 2013), we conclude that the strong FliS-FlhA_C_ interaction allows the PMF-driven export gate complex to efficiently unfold FliC monomer for its export as suggested before (Furukawa et al., 2016).

### Effect of FliS Depletion on the Elongation of Flagellar Filament

**Figure 5:**
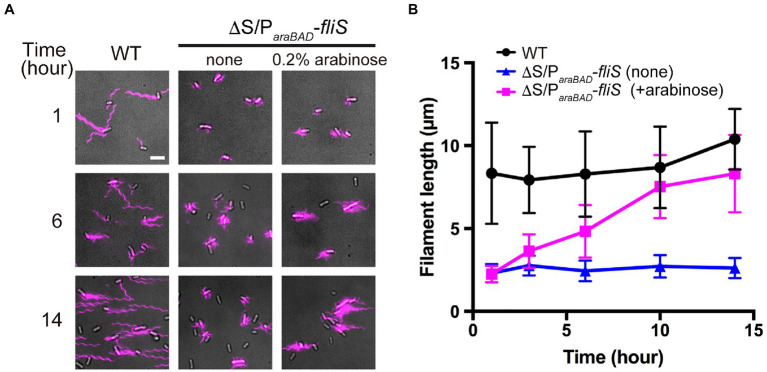
Effect of FliS expression on filament growth. **(A)** Fluorescent images of ∆*fliS* ∆*araBAD*::*fliS* (∆S/P*_araBAD_*-*fliS*) cells. The ∆S/P*_araBAD_*-*fliS* cells were exponentially grown at 30°C to express and accumulate FliC subunits in the cytoplasm, and then, arabinose was added at a final concentration of 0.2%(w/v) to induce the expression of FliS from the *araBAD* promoter on the chromosome. Cells were collected at the indicated time intervals, followed by labelling their flagellar filaments with a fluorescent dye. Wild-type cell (WT) was used as the positive control. Scale bar, 5.0μm. **(B)** Average length of flagellar filaments. Filament length is the average of 50 filaments, and vertical lines are standard deviations (also see Supplementary Table 2).

To investigate when the PMF-driven export gate complex requires FliS for efficient unfolding process of FliC monomer during filament assembly, we constructed a *Salmonella* ∆S ∆*araBAD*::*fliS* strain (thereafter referred to as ∆S/P*_araBAD_*-*fliS*), in which FliS is expressed from an arabinose-inducible P*_araBAD_* promoter at the *araBAD* locus on the chromosome. The ∆S/P*_araBAD_*-*fliS* cells were exponentially grown at 30°C to express and accumulate FliC subunits in the cytoplasm, and then, arabinose was added at a final concentration of 0.2% (w/v) to express FliS. We collected cell samples at regular time intervals, labelled their flagellar filaments with a fluorescent dye (Figure 5A), and measured the filament length (Figure 5B and Supplementary Table 2). When FliS was expressed by adding arabinose, the average flagellar filament length gradually increased with the culture time and reached almost the wild-type length. In contrast, when FliS was not expressed, the filament length remained at about 2.5μm even after a prolonged incubation time. These results suggest that the export gate complex can transport FliC molecules into the cell exterior in a FliS-independent manner in the early stage of filament assembly and that FliS assists efficient unfolding and transport of FliC molecules by the export gate complex to produce long flagellar filaments.

### Effect of FliS Depletion on the Secretion of FlgM and FliC in the Early Stage of Filament Assembly

**Figure 6:**
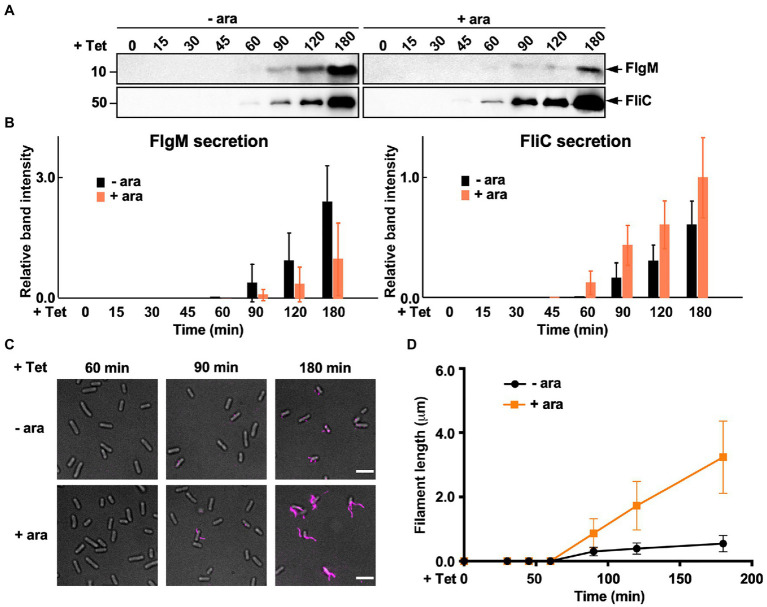
Measurements of the secretion rates of FlgM and FliC using ∆S/P*_araBAD_*-*fliS* cells containing a T-pop insertion. **(A)** Immunoblotting, using polyclonal anti-FlgM (upper panel) or anti-FliC (lower panel) antibody, of heat-treated cultures of ∆S/P*_araBAD_*-*fliS* T-pop cells. The ∆S/P*_araBAD_*-*fliS* T-pop cells were grown at 30°C in L-broth until the cell density reached an OD_600_ of 0.5. After adding tetracycline with (+ara) or without (−ara) arabinose, cell cultures were collected at 0, 15, 30, 45, 60, 90, 120, and 180min and heated at 65°C for 5min to depolymerize flagellar filaments into FliC monomers. Molecular mass markers are indicated on the left. **(B)** Relative extracellular levels of FlgM (left panel) and FliC (right panel). The density of each FlgM or FliC band on immunoblots was normalized for the level of FlgM and FliC in FliS^+^ cells at 180min. These data were the average of four independent experiments. Vertical bars indicate standard deviations. **(C)** Fluorescent images of the ∆S/P*_araBAD_*-*fliS* T-pop cells. The cells were exponentially grown in L-broth. After adding tetracycline with or without arabinose, the cells were collected at the indicated time intervals, followed by labelling their flagellar filaments with a fluorescent dye. **(D)** Average length of flagellar filaments. Filament length was the average of 50 filaments. Vertical lines indicate standard deviations (also see Supplementary Table 3).

During HBB assembly, FlgM binds to FliA to inhibit its σ^28^ activity (Ohnishi et al., 1992). Upon completion of hook assembly, the fT3SS switches the rod- and hook-type mode to the filament-type mode and starts secreting FlgM into the culture media (Figure 1). As a result, FliA becomes σ^28^ to induce the transcription of the *fliC* gene (Hughes et al., 1993; Kutsukake, 1994). FliS also binds to FlgM to suppress FlgM secretion during filament assembly to avoid undesirable over-expression of class 3 genes (Yokoseki et al., 1996; Galeva et al., 2014; Xu et al., 2014). Because FliS is expressed from the class 2 promoter of the *fliDST* operon during HBB assembly (Figure 1; Chevance and Hughes, 2008), we hypothesized that FliS may function as a negative regulator to inhibit FlgM secretion upon onset of filament formation and then may become an export chaperone somewhat later to escort FliC to the FlhA_C_ ring. Because precise measurement of the export rate of flagellar building blocks requires the external onset control of flagellar gene expression, we constructed the ∆S/P*_araBAD_*-*fliS* strain containing T-POP, in which FlhD and FlhC are expressed from a tetracycline-inducible promoter only in the presence of tetracycline (Karlinsey et al., 2000b). The ∆S/P*_araBAD_*-*fliS* T-POP cells were grown at 30°C in L-broth until OD_600_ reached *ca*. 0.4–0.6. After adding tetracycline with or without arabinose, the cells were collected at regular time intervals and heated at 65°C for 5min to prepare total extracellular FliC molecules (FliC polymerized into the filaments attached to *Salmonella* cell bodies and FliC monomers secreted into the culture media) as well as extracellular FlgM molecules, followed by immunoblotting with polyclonal anti-FlgM or FliC antibody to measure the amounts of FlgM and FliC (Figure 6A). When FliS was expressed by adding arabinose, the level of FlgM secretion was lower than that in the absence of FliS (Figure 6A, upper panels and Figure 6B, left panel). In contrast, the secretion level of FliC was higher when FliS was expressed (Figure 6A, lower panels and Figure 6B, right panel). Consistently, the filament growth, which started at around 60min after tetracycline induction, showed a steady elongation rate to form long filaments in the presence of FliS but was much slower and retarded at markedly shorter lengths in the absence of FliS (Figures 6C,D and Supplementary Table 3). These results suggest that the PMF-driven export gate complex requires FliS to facilitate unfolding and transport of FliC for rapid growth of the filament to form long ones for high level motility.

## Discussion

Flagellar filament assembly begins with the assembly of the hook-filament junction at the hook tip, followed by the filament cap and finally the filament with the help of the filament cap (Figure 1). The flagellar export chaperones facilitate the docking of their cognate filament-type substrates to the FlhA_C_ ring, thereby allowing the fT3SS to efficiently transport the substrates into the central channel of the growing flagellar structure (Bange et al., 2010; Minamino et al., 2012a; Kinoshita et al., 2013; Furukawa et al., 2016). FlgN and FliT require the cytoplasmic ATPase complex consisting of FliH, FliI, and FliJ to efficiently bind to the FlhA_C_ ring, whereas FliS does not (Thomas et al., 2004; Evans et al., 2006; Bange et al., 2010; Sajó et al., 2014; Minamino et al., 2016; Inoue et al., 2018). These observations lead to a plausible hypothesis that such differences in the binding affinity of flagellar chaperones for the cytoplasmic ATPase complex contribute to the ordered export of their cognate substrates for efficient formation and growth of the flagellar filament after hook assembly. Because FliS does not bind to the cytoplasmic ATPase complex (Sajó et al., 2014), it raises an interesting question of how FliC subunit is unfolded?

To clarify how the flagellar export chaperones facilitate the docking of their cognate substrates to FlhA_C_, which is followed by subsequent unfolding and transport of the export substrates by the fT3SS, we constructed the ∆NS, ∆NT, and ∆NST mutants and found that deletion of either FliS, FliT, or both increased the expression levels of FlgK and FlgL, thereby significantly increasing the probability of assembling the hook-filament junction at the hook tip even in the absence of FlgN (Figure 2). The ∆NS, ∆NT, and ∆NST mutants still leaked a large amount of unassembled FliC monomers into the culture media (Figures 2D,E,I). Furthermore, the ∆S and ∆T mutants also leaked more FliC monomers into the culture media than the WT (Figures 2F,G). These results indicate that FlgN, FliT, and FliS all prevent unassembled FliC monomers from leaking out into the culture media during filament assembly. Therefore, we conclude that the binding of these flagellar chaperones to FlhA_C_ is required for efficient and robust filament formation at the hook tip.

**Figure 7:**
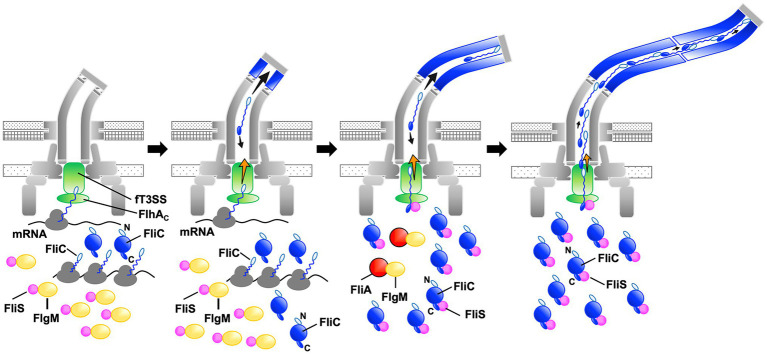
Model for processive filament assembly at the hook tip. Flagellar filament assembly is composed of two distinct, FliS-independent and FliS-dependent processes. Upon completion of the hook structure, FlgM is secreted *via* the flagellar type III secretion system (fT3SS) into the culture media, thereby allowing FliA to act as a flagellum-specific sigma factor to drive the transcription of the *fliC* gene. As a result, a large amount of FliC is accumulated in the cytoplasm. FliC monomers are then unfolded and injected into the central channel of the flagellum by fT3SS one after another and transported to the distal end by diffusion to start forming the filament. Upon onset of this filament assembly, FliS binds to FlgM to inhibit FlgM secretion, and so FliC secretion and flagellar filament formation occur in a FliS-independent manner (left and left middle panels, steps 1 and 2). Then, FliS transfers FlgM to FliA to avoid the over-expression of FliC during filament assembly and becomes an export chaperone to assist FliC unfolding and export by fT3SS. FliS binds to the extreme C-terminal region of FliC with high affinity and facilitates FliC docking to the FlhA_C_ ring. FliS also supports the fT3SS to efficiently unfold and inject FliC subunits into the central channel of the flagellum, which is followed by their diffusion down to the distal end of the growing structure where they assemble to form the filament (middle right, step 3). At the beginning of filament assembly, the distance from the fT3SS export gate to the assembly point is short, and the time for unfolded FliC monomers to reach the distal end is relatively short. The central channel may not be so crowded by FliC monomers, and therefore, the fT3SS export gate may not require such strong force to unfold and inject FliC into the central channel as it requires later when the filament becomes longer. FliS-assisted FliC unfolding would not be necessary in this stage when the filament is short (middle right, step 3). As the filament elongates, however, the diffusion time progressively increases, making the central channel very crowded (right, step 4). The fT3SS export gate now requires much stronger force for FliC unfolding and injection to continue filament growth, and FliS-assisted unfolding of FliC becomes essential in this later stage of filament assembly. That is why FliS deletion stops filament growth at relatively short lengths.

The transmembrane export gate complex utilizes PMF across the cytoplasmic membrane to facilitate protein unfolding and injection into the central channel of the growing structure with a diameter of about 1.3nm (Minamino and Namba, 2008; Paul et al., 2008; Fujii et al., 2017). This has been verified by *in vitro* reconstitution experiments using inverted membrane vesicles (Terashima et al., 2018, 2020). To investigate why the ∆NST mutant produces short flagellar filaments (Figure 3), we isolated pseudorevertants from the ∆NST mutant and found that all suppressor mutations are located in the *fliC* gene (Figure 4). FliS binds not only to the extreme C-terminal region of FliC but also to domain D1 of FliC (Altegoer et al., 2018). It has been shown that the R92S and Q128R substitutions in domain D1 of FliC and deletion of domain D3 destabilize the folded structure of FliC monomer (Furukawa et al., 2016). We also confirmed that FliS is required for efficient FliC export for elongation of the filament beyond a certain length around 2.5μm (Figure 5). Because FliS itself does not have the unfoldase activity (Furukawa et al., 2016), we suggest that FliS must be assisting the unfolding process of FliC by the PMF-driven export gate complex and, in the absence of FliS, the rate of FliC export is significantly limited by a lower rate of FliC unfolding by the export gate complex alone. Because the export of flagellar building blocks is not obligatorily coupled to protein translation (Hirano et al., 2003; Terashima et al., 2018, 2020), we suggest that an interaction between FliS and FlhA_C_ becomes essential for efficient unfolding and transport of FliC by the export gate complex to form long filaments after FliC synthesis is complete (Figure 7).

Why can *Salmonella* cells lacking FliS produce short filaments? FliS binds to FlgM to prevent its secretion *via* the fT3SS into the culture media during filament assembly. As a result, FlgM becomes the anti-sigma factor again to suppress the σ^28^ activity of FliA to control the number of flagella per cell (Yokoseki et al., 1996; Galeva et al., 2014; Xu et al., 2014). In this study, we analyzed the secretion rates of FlgM and FliC in the early stage of filament assembly and found that FliS suppressed FlgM secretion and facilitated FliC secretion, thereby allowing cells expressing FliS to produce long flagellar filaments (Figure 6). The filament length of cells lacking FliS remained about 2.5μm even after a prolonged incubation time (Figure 5). Thus, it seems likely that the fT3SS can transport FliC molecules in a FliS-independent manner for a short period after onset of filament formation, and that is why the cells lacking FliS can produce short flagellar filaments. Because it has been reported that the untranslated region of mRNA around the start codon of the *fliC* gene is involved in the targeting of FliC molecules to the fT3SS (Singer et al., 2014), we propose that FliS-independent FliC export may occur in a co-translational manner when FliS is busy inhibiting FlgM secretion (Figure 7).

Why does the filament growth stop in the absence of FliS although a large amount of FliC is expressed in the cytoplasm? The filament elongation rate is determined as the sum of the PMF-driven injection rate of FliC monomer by the fT3SS at the flagellar base and the diffusion rate of FliC subunit inside the long and narrow channel along the length of the flagellum (Chen et al., 2017; Renault et al., 2017). In the early stage of filament assembly, the distance from the export gate to the assembly point is short, and so the diffusion time is short. As a result, the PMF-driven unfolding and injection steps of FliC by the fT3SS are the rate-limiting in the filament growth, and each FliC subunit reaches its assembly point without encountering any other subunits (Figure 7). As the filament elongates, the diffusion time progressively increases, and it eventually becomes comparable to or longer than the unfolding and injection time. At this stage, the central channel of the growing filament would be crowded with FliC subunits, thereby decreasing the diffusion rate (Figure 7). The export gate would then need a stronger force to unfold and inject FliC monomers or some assistance in the unfolding process to keep the effective injection rate for continued filament growth. Because FliC mutations that destabilized the folded structure of FliC monomer allowed the cells lacking FliS to produce long filaments (Figure 4), we propose that the PMF-driven export gate complex requires the interaction of FliS with FlhA_C_ to facilitate efficient and rapid unfolding and injection of FliC at the flagellar base so that the newly injected FliC subunit can push many unfolded FliC subunits crowded in the long central channel to the distal growing end for continued elongation of the long filament (Figure 7). This indicates that the efficient growth of the long flagellar filament is achieved by a delicate balance between the PMF-driven FliC unfolding and injection force at the export gate and the diffusion rate of unfolded FliC monomers crowded in the narrow and long central channel of the filament.

The assembly of FliC subunits into the filament occurs by a template-structure-driven mechanism. Both intrinsically disordered N- and C-terminal segments of FliC form domain D0 in the innermost core of the filament when FliC monomers polymerize into the filament structure with the help of the FliD cap. Intermolecular interactions between the D0 domains of FliC subunits and those between domain D0 of FliC and domain D0 of FliD are required for filament assembly (Yonekura et al., 2003; Al-Otaibi et al., 2020). Even though FlgN and FliT are present, the ∆S mutant leaked more FliC monomers out into the culture media than the WT cells (Figure 2F). This raises the possibility that FliS may affect the local conformation of the N- and/or C-terminal segments of FliC, which in turn affects the proper folding of other domains to make it easier for the PMF-driven export gate complex to unfold the entire FliC molecule for its efficient export for filament assembly.

## Data Availability Statement

The original contributions presented in the study are included in the article/**Supplementary Material**, further inquiries can be directed to the corresponding author.

## Author Contributions

TM and KN conceived and designed the research and wrote the paper based on discussion with YM and MK. TM, MK, and YM performed the experiments and analyzed the data. All authors contributed to the article and approved the submitted version.

## Funding

This work was supported in part by JSPS KAKENHI Grant Numbers JP26293097 and JP19H03182 (to TM), JP18K14638 and JP20K15749 (to MK), JP18K06159 and JP21K06099 (to YM) and JP25000013 (to KN), and MEXT KAKENHI Grant Numbers JP15H01640 and JP20H05532 (to TM), and JST PRESTO Grant Number JPMJPR204B (to YM). This work has also been partially supported by JEOL YOKOGUSHI Research Alliance Laboratories of Osaka University to KN.

## Conflict of Interest

The authors declare that the research was conducted in the absence of any commercial or financial relationships that could be construed as a potential conflict of interest.

## Publisher’s Note

All claims expressed in this article are solely those of the authors and do not necessarily represent those of their affiliated organizations, or those of the publisher, the editors and the reviewers. Any product that may be evaluated in this article, or claim that may be made by its manufacturer, is not guaranteed or endorsed by the publisher.
