# Supplementary material for: Multiple Roles of Flagellar Export Chaperones for Efficient and Robust Flagellar Filament Formation in Salmonella
Source: Front Microbiol. 2021 Oct 6;12:756044. doi: 10.3389/fmicb.2021.756044 (PMC8527033; doi:10.3389/fmicb.2021.756044)
Supplement: Supplementary file 1 [file Data_Sheet_1.PDF]

Supplementary Information

Multiple roles of flagellar export chaperones for efficient and robust flagellar filament formation in *Salmonella*

Tohru Minamino, Yusuke V. Morimoto,

Miki Kinoshita and Keiichi Namba

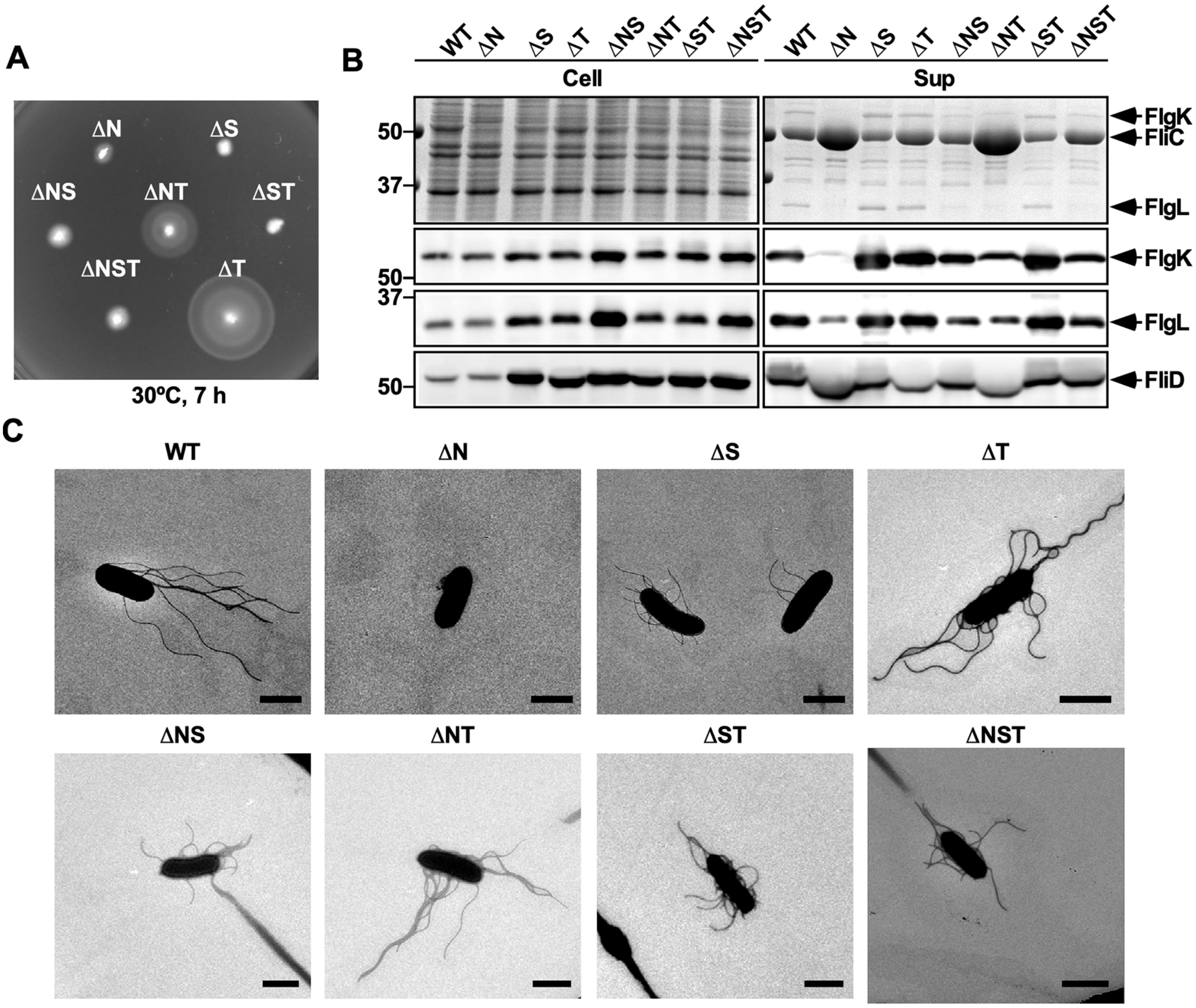

Supplementary Figure 1. Characterization of chaperone-deficient mutants. (A) Motility of *Salmonella* Δ*flgN* (ΔN), Δ*fliS* (ΔS), Δ*flgN* Δ*fliS* (ΔNS), Δ*flgN* Δ*fliT* (ΔNT), Δ*fliS* Δ*fliT* (ΔST), Δ*flgN* Δ*fliS* Δ*fliT* (ΔNST) and Δ*fliT* (ΔT) mutants. Plates were incubated at 30ºC for 7 hours. (B) Flagellar protein export properties. The above *Salmonella* mutants were exponentially grown in L-broth, and then the cultures were heated at 65ºC for 5 min. After centrifugation, the cell pellets (Cell) and supernatants (Sup) were prepared separately. After SDS-PAGE, protein samples were analyzed by CBB staining (1st row) and immunoblotting, using polyclonal anti-FlgK (2nd row), anti-FlgL (3rd row) or anti-FliD (4th row) antibody. Molecular size markers (kDa) are indicated on the left. (C) Electron micrographs of the above cells. Cells were exponentially grown in L-broth and then negatively stained with 0.5% (w/v) phosphotungstic acid, pH 6.5. Micrographs were taken at a magnification of ×1,200. Scale bar, 2 μm.

A

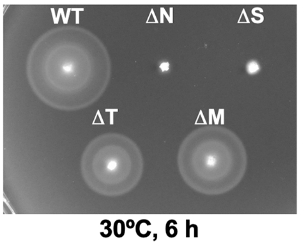

B

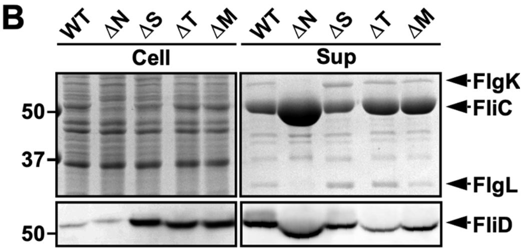

Supplementary Figure 2. Effect of deletion of FliT on FliD export. (A) Motility of the *Salmonella* wild-type (WT), Δ*flgN* (ΔN), Δ*fliS* (ΔS), Δ*fliT* (ΔT) and Δ*fliM* (ΔM) strains. Plates were incubated at 30ºC for 6 hours. (B) Flagellar protein export properties. The above *Salmonella* mutants were exponentially grown in L-broth, and then the cultures were heated at 65ºC for 5 min. After low-speed centrifugation, the cell pellets (Cell) and supernatants (Sup) were prepared separately. After SDS-PAGE, protein samples were analyzed by CBB staining (1st row) and immunoblotting using polyclonal anti-FliD (2nd row) antibody. Molecular size markers (kDa) are indicated on the left.

Supplementary Table 1. Effect of FliS deletion on average length of flagellar filaments

Strain	Fraction of flagellated cells (%)	Average filament length (µm) (mean ± SD)
WT	100 (n = 122)	8.9 ± 1.9 (n = 50)
∆NST	85.1 (n = 134)	2.8 ± 0.8 (n = 50)
∆NST *fliC*(Q128R)	78.5 (n = 65)	5.6 ± 1.3 (n = 50)
∆S	90.0 (n = 130)	3.0 ± 0.7 (n = 50)

Supplementary Table 2. Effect of FliS expression on average length of flagellar filaments

	Average filament length (μm)<br>(mean ± SD)		
Time (hour)	WT	ΔS/P_{araBAD}-*fliS*	
		none	0.2% arabinose
1	8.3 ± 3.0<br>(n = 50)	2.3 ± 0.5<br>(n = 50)	2.3 ± 0.6<br>(n = 50)
3	7.9 ± 2.0<br>(n = 50)	3.6 ± 1.0<br>(n = 50)	2.8 ± 0.6<br>(n = 50)
6	8.3 ± 2.6<br>(n = 50)	4.8 ± 1.6<br>(n = 50)	2.4 ± 0.6<br>(n = 50)
10	8.7 ± 2.5<br>(n = 50)	7.5 ± 1.9<br>(n = 50)	2.7 ± 0.7<br>(n = 50)
14	10.4 ± 1.8<br>(n = 50)	8.3 ± 2.3<br>(n = 50)	2.6 ± 0.6<br>(n = 50)

Supplementary Table 3. Effect of FliS expression on average length of flagellar filaments during the early stages of filament assembly

	Average filament length (μm)<br>(mean ± SD)	
Time (min)	ΔS/P$_{araBAD}$-*fliS* T-POP	
	none	0.2% arabinose
0	–	–
30	–	–
45	–	–
60	–	–
90	0.3 ± 0.1<br>(n = 50)	0.9 ± 0.5<br>(n = 50)
120	0.4 ± 0.2<br>(n = 50)	1.7 ± 0.8<br>(n = 50)
180	0.5 ± 0.3<br>(n = 50)	3.2 ± 1.1<br>(n = 50)

–, no detectable flagellar filament.
